# Academic Procrastination in Children and Adolescents: A Scoping Review

**DOI:** 10.3390/children10061016

**Published:** 2023-06-05

**Authors:** Marcela Paz González-Brignardello, Angeles Sánchez-Elvira Paniagua, M. Ángeles López-González

**Affiliations:** 1 Department of Personality Psychology, Psychological Assessment and Treatment, Faculty of Psychology, Universidad Nacional de Educación a Distancia, UNED, 28040 Madrid, Spain; mpgonzalez@psi.uned.es (M.P.G.-B.); asanchez-elvira@psi.uned.es (A.S.-E.P.); 2 Psychology Department, Faculty of Health Sciences, Universidad Rey Juan Carlos, 28922 Madrid, Spain

**Keywords:** academic procrastination, student procrastination, children, adolescents, school, scoping review

## Abstract

Academic procrastination is a persistent behavior in students’ academic development consisting of postponing or delaying the completion of necessary tasks and having a deadline for completion, which is associated with detriment in performance, school dropout, and loss of student well-being. The largest body of existing knowledge on this behavior comes from studies conducted with university students, although it is necessary to deepen the findings obtained at lower educational levels. The aim of this work has been to carry out a scoping review of the empirical publications focused on academic procrastination in children and adolescents. The inclusion and exclusion criteria are detailed following the general guidelines of the Joanna Briggs Institute. However, some modifications are incorporated in the flowchart to guide the review sequence. The search was conducted in eleven thematic (ERIC, MedLine, Psychology and Behavioral Sciences Collection, PsycINFO, PubPsych, and Teacher Reference Center) and multidisciplinary databases (Academic Search Ultimate, E-Journals, ProQuest, Scopus, and Web of Science) to identify relevant publications up to 2022, including grey literature. Out of the initial 1185 records screened, a total of 79 records were selected. The search results included a total of 79 records. The most used assessment instruments, the most studied variables, and the type of design and sources of information used in the selected studies are detailed. Cultural aspects that open new lines of future research are identified.

## 1. Introduction

Procrastination is a very common and pervasive behavior in different areas of human activity. It involves the intentional delay of actions and behaviors that have a time limit within which they should be completed [1,2]. There is a tendency to assume that everyone procrastinates or delays some necessary activity (e.g., medical appointments, exercise, paying fines, going to bed, among many other possible activities). However, to the extent that this behavior occurs on a frequent basis, it has consequences for those who engage in it, and may even affect others if it occurs, for example, in work or collaborative learning environments. Contrary to popular belief, people who engage in this behavior have a desire to complete tasks or actions, but have difficulty in translating these intentions into implementation actions, initiation, and completion [3].

Procrastination is a complex, poorly understood behavior that involves different cognitive, emotional, and behavioral components [4,5,6]. It has been understood as a failure of self-regulation [4], an avoidance behavior toward unpleasant tasks [7], due to fear of failure [8], fear of success [9], or an expression of poor action control [9], and it has been consistently associated with low self-efficacy, e.g., [10].

Academic procrastination, a type of domain-specific behavior, refers to the tendency of students to delay or postpone completing academic tasks, such as studying for an exam, doing homework, or writing an essay, even though they know they should perform these actions and have a specific deadline for completion. Academic procrastination has garnered significant attention from researchers, primarily due to two factors: (a) Its high prevalence among university students and, on the other hand, (b) the ease of access to samples of students. Approximately 80% of college students are estimated to procrastinate, making it one of the most prevalent issues among post-secondary students, with estimates ranging from 10% to 70% [11,12]. Contrary to the previous statement, little research has been conducted to understand the characteristics of procrastination in younger age groups, e.g., [13]. However, researchers became interested in studying the behavior of children and adolescents during the prolonged periods of confinement due to the COVID-19 pandemic. Research in relation to procrastination focused primarily on the significant increase in the use of electronic devices and social media, as well as the procrastination of academic activities that were mandatory online during that period, e.g., [14,15,16].

Academic procrastination leads to a decline in students’ well-being. It has been associated with poor academic performance [17], emotional distress (stress, anxiety, and depression) [18,19,20], and physical health deterioration, e.g., [21,22].

One of the many unanswered questions regarding academic procrastination pertains to its development in students. Is academic procrastination a behavioral pattern that develops at an early age, or does it emerge as a reaction or response as students face the transition to university level? If the behavior does develop at an early age, what is its prevalence in primary and secondary education, what role do parents and the education system play in the development of the problem, and are there interventions to change children’s procrastinating behavior? If so, what factors are involved in promoting change?

**Figure 1:**
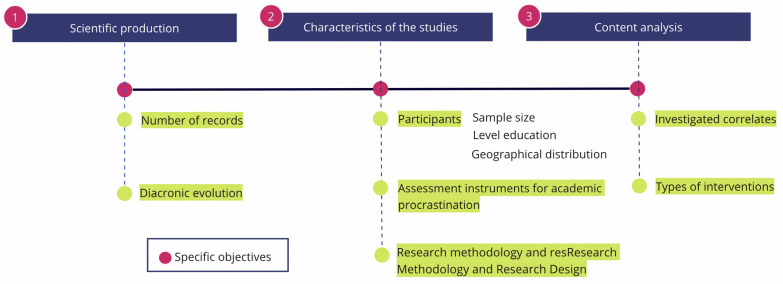
Flowchart of the specific objectives.

The aim of this work has been to carry out a panoramic review of the empirical publications focused on academic procrastination in children and adolescents. We propose a scoping review that will also allow us: (a) To identify the production and evolution of publications on academic procrastination in primary and secondary education; (b) to specify the methodological basic characteristics of the studies; (c) to conduct a content analysis to categorize the correlates investigated in relation to academic procrastination in this age group and to determine the types of interventions reported (see Figure 1). The ultimate goal, which is the essence of a systematic scoping review, is to detect gaps in research to contribute to future lines of inquiry.

## 2. Method

Following the methodology implemented in previous studies [23,24], this review includes several control mechanisms designed to reduce any bias that may exist a priori, as suggested by the PRISMA-ScR [25] (see Appendix A) and the JBI Evidence Synthesis Manual [26]. The study was registered in the international registry for overview reviews and the protocol is available from OSF if required (https://doi.org/10.17605/OSF.IO/KCXJ9, accessed on 11 May 2023). Therefore, we developed a protocol that has allowed for uniform criteria to be applied to each of the registries, from the initial search for papers to the inclusion of the final papers.

### 2.1. Inclusion and Exclusion Criteria

Based on the research question and following an adaptation of the PICO strategy, a protocol called “d-Cocospe” (documents, concept, context, studies, participants, and evaluation) was designed following the indications of various authors, e.g., [27] (see Table 1). 

#### 2.1.1. Documents

Both periodical (journal articles) and non-periodical (books, book chapters, and doctoral theses) publications were included. Magazine articles, editorials, conferences, and other similar types of documents were excluded. 

#### 2.1.2. Concept

The focus of the study was on publications in which academic procrastination was assessed.

#### 2.1.3. Context

The study focused specifically on publications that assessed academic procrastination within an academic context. As a result, publications on general procrastination or on procrastination in other contexts (such as work, health, or leisure activities) were excluded.

#### 2.1.4. Studies

Empirical studies were included and theoretical studies, literature reviews, and case studies were not considered.

#### 2.1.5. Participants

The study subjects were exclusively students under 18 years and publications that included university students, community samples or those that did not specify the age or educational level of the participants were discarded. 

#### 2.1.6. Evaluation

Empirical studies in which the sample size and mode of assessment of procrastination was explicitly stated were included, if procrastination was assessed by behavioral or reported procrastination measures (i.e., self-reports or hetero reports). On the other hand, publications where procrastination was assessed by single-item instruments or unstructured instruments were excluded.

### 2.2. Search Strategy

The search equation was developed by the authors (MPGB, ASEP, and MALG) through initial exploratory searches in collaboration with expert documentalists from the Universidad Nacional de Educación a Distancia (UNED). 

The final search was constructed by applying Boolean operators (AND & OR) and truncation (* and inverted commas) in TI, AB, KW: (“academic procrast*” OR “student procrast*”) AND (child* OR adolesc* OR young OR teen* OR school OR infan* OR boy* OR girl* OR junior OR kid)] including publications up to December 2022. In the ProQuest database, NOFT was used: [(“academic procrast*” OR “student procrast*”) AND (child* OR adolesc* OR young OR teen* OR school OR infan* OR boy* OR girl* OR junior OR kid)]. For WoS, the search was executed in TOPIC: [(“academic procrast*” OR “student procrast*”) AND (child* OR adolesc* OR young OR teen* OR school OR infan* OR boy* OR girl* OR junior OR kid)] including publications up to December 2022.

### 2.3. Sources of Information

The documentary search was carried out in July 2022. Subsequently, in March 2023, the references were updated, and the records found up to December 2022 were included to replicate the search and incorporate new records.

#### 2.3.1. Formal Strategies

The records were obtained through different sources of information. First, 11 automated databases were consulted: (a) Thematic databases in the areas of Psychology and Education: ERIC, MedLine, Psychology and Behavioral Sciences Collection, PsycINFO, PubPsych, and Teacher Reference Center, and (b) multidisciplinary: Academic Search Ultimate, E-Journals, ProQuest, Scopus, and Web of Science. The search was conducted without language restrictions.

Second, the search was complemented by consulting documents located in different international repositories, such as Redined, Cogprints, Zenodo, BASE, NDLTD Networked Digital Library of Theses and Dissertations, OAIster, and arxiv. Bibliographic references were also analyzed to identify possible publications.

#### 2.3.2. Informal Strategies

Academic social networks (e.g., ResearchGate, academia.edu, Dimensions, etc.) were also explored to locate publications by relevant researchers in the field.

#### 2.3.3. Retrospective Strategies

The search was complemented by analysis of systematic reviews and meta-analyses to retrieve potentially relevant articles.

The searches were conducted without applying language restrictions to control for possible linguistic bias. In this regard, online translation (DeepL and Google Translator) of the original documents was used when necessary (e.g., texts in Turkish, Farsi, Chinese, Indonesian, etc.).

### 2.4. Coding and Identification of Records and Data Extraction

All records obtained from each database were exported to separate libraries in the EndNote 20.5 software package. EndNote 20.5 allowed for the files to be merged into a single library, which facilitated the removal of duplicate items. The records were then exported to a shared spreadsheet in Google Drive, in order that the authors could work collaboratively. After several online meetings, a protocol was established to create the analysis fields for processing each record (data charting). Then, a series of bibliometric data were included: (a) Year of publication; (b) authorship; (c) title; (d) journal or book name; (e) DOI; and (f) abstract. In addition, several enriched fields were added in accordance with the d-Cocospe format: (a) Document typology: Journal articles, books, book chapters, theses, etc.; (b) Concept: It was specified whether the document dealt with procrastination. If this concept was not attended, the topic of analysis was specified; (c) Context: In each record it was indicated whether procrastination was confined to the academic field, otherwise, the specific context was indicated; (d) Study: The type of study was indicated, specifying whether it was an empirical study, theoretical study, review or case study; (e) Participants: The following information was collected: (i) Number of participants in the study; (ii) type of participants: Students, general population, young volunteers, etc.; (iii) education level: Primary education (children aged 6–7 to 11–12) or secondary education (students aged 12 to 17–18); (iv) educational grade or rank; and (v) geographical origin of the sample; (f) Evaluation: Information on the assessment instrument used to measure academic procrastination was included; (g) Type of design: Experimental, quasi-experimental, ex post facto or observational; (h) Type of intervention: Content of the interventions, type of format (individual vs. group), intervention setting, structure of each session, and record of measures taken; (i) Analyzed variables: The variables analyzed were categorized according to different domains: Sociodemographic domain, learning domain, health domain, relational domain, and intrapersonal domain; (j) Outcomes; and (k) Conclusions in relation to academic procrastination.

During the coding and data extraction process, regular meetings were held to discuss inconsistencies, doubts, and disagreements. Each reference was independently analyzed by two researchers (MPGB and MALG), and both relevant and non-relevant records were recorded, in order that all the items were coded after reading the title, abstract, and full text. 

The next phase of the review consisted of leading two types of analysis: (a) Thematic content analysis of the variables, and (b) classification of experimental interventions based on the analysis of the variables under study. 

To carry out the thematic content analysis, a sequential procedure was followed: (a) Generation of a list of variables measured in the documents’ titles, abstracts, and full texts; (b) configuration of initial categories (a posteriori) through inductive analysis; (c) creation of a category tree, grouping them into conceptual families; and (d) re-labeling and reducing categories to a more inclusive level.

The intervention classification was carried out by categorizing the fields where the intervention was directed. Then, for each study, the type of technique or program implemented was collected. The type of design, variables, and most relevant results obtained were recorded.

Finally, we present the results using summary tables and figures created with Microsoft Excel (version 16.7), MapChart, MIRO (online whiteboard), and Infogram, along with a narrative description of the main findings.

## 3. Results

### 3.1. Production and Evolution of Publications

**Figure 2:**
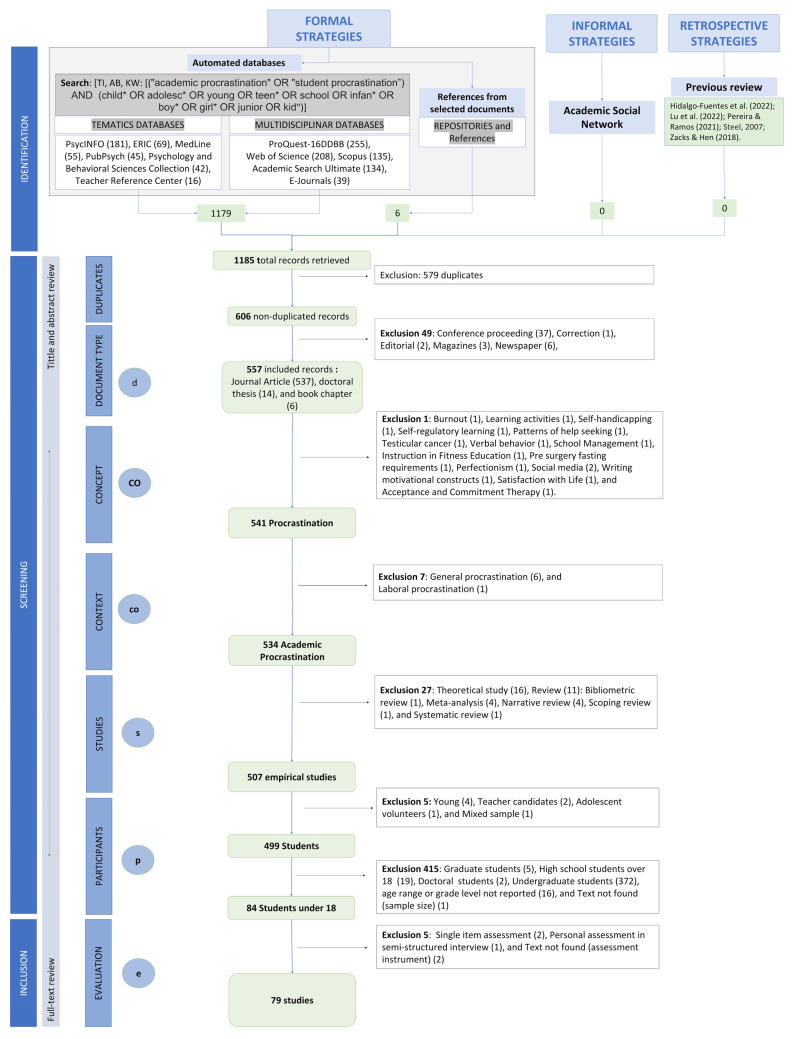
Flowchart of the review process. The literature search has been conducted using formal strategies (automated databases and references from selected documents), informal strategies (academic social networks) and retrospective strategies [4,28,29,30,31].

The search strategies allowed us to identify 1185 documents, of which 79 met the criteria for inclusion, representing 6.67% of the initial records. Figure 2 shows the entire process carried out from the selection of formal and informal strategies used, as well as the number of records and the reasons for inclusion and exclusion of each document in each phase of the screening process.

**Figure 3:**
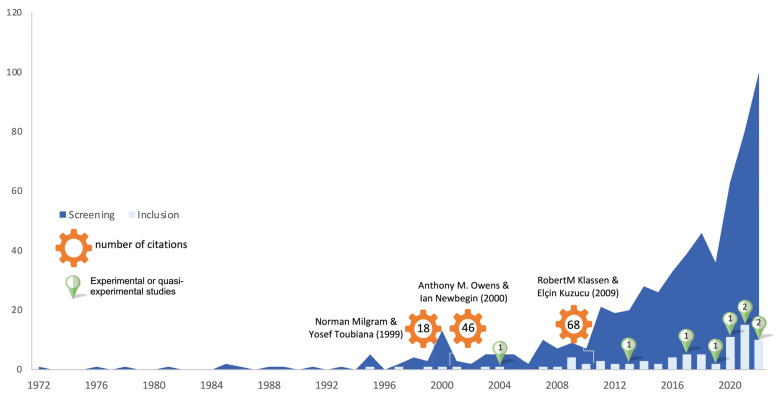
Diachronic evolution of the literature on academic procrastination in children and adolescents. In this graphic, it have been indicated the most cited refs. [32,33,34].

On the other hand, Figure 3 shows the diachronic evolution of publications on academic procrastination in students under 18 years of age. The first selected work was published in 1995, although the evolution of publications has been irregular until the last 15 years, with the peak of records in 2021 with 15 documents.

A comparison is made between the initially retrieved publications and the documents finally included. Similarly, the three publications with the highest number of citations in the Web of Science database in 2023 are labeled and the only experimental studies conducted to date are marked.

### 3.2. Characteristics of the Studies

Using the prototypical structure of the “Methods” section of any scientific publication, the characteristics of the studies are presented below: Participants, assessment, and type of design (see Appendix B).

#### 3.2.1. Participants

**Figure 4:**
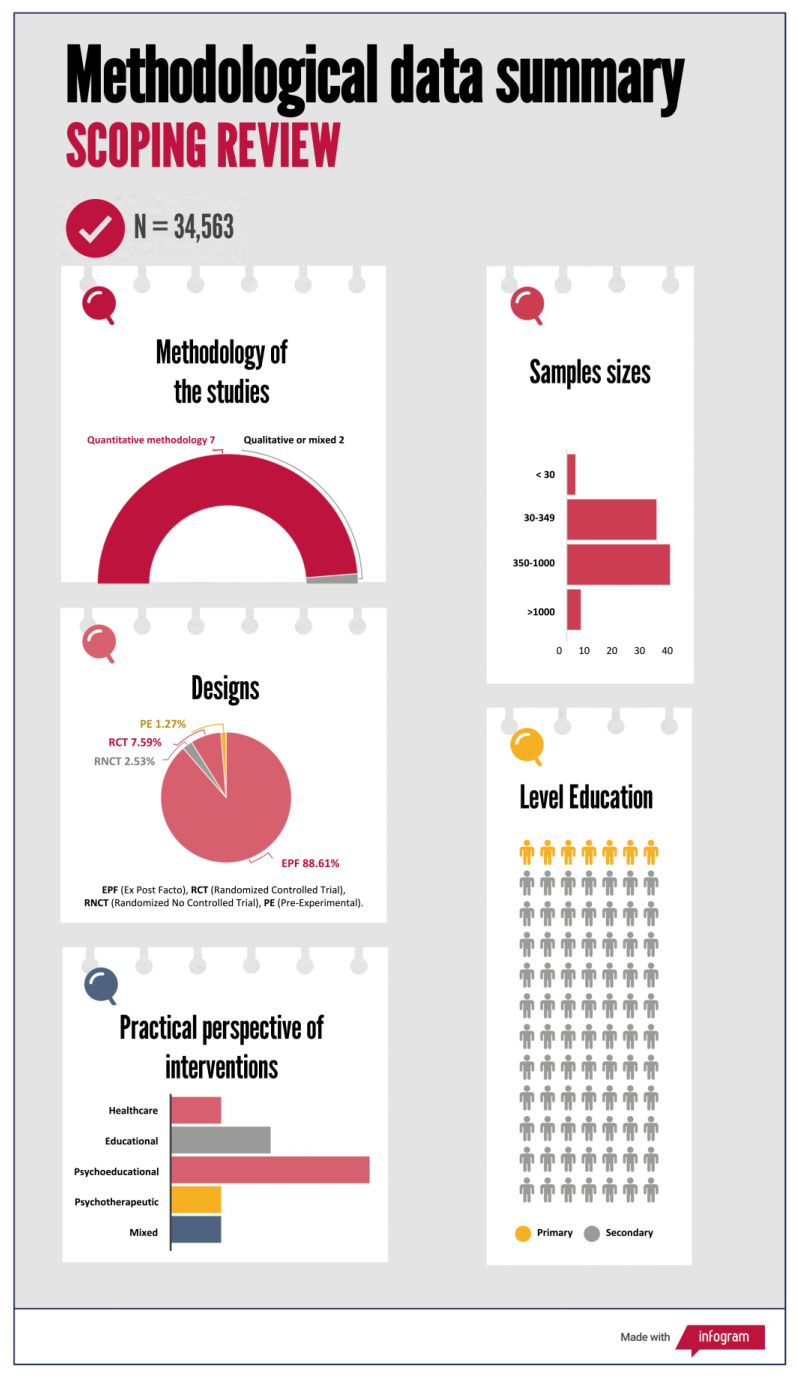
Infographic: Summary of study methodology.

In terms of sample size, a total of 34,563 participants were counted, with an average per study equal to 437.5 students (min. = 5 and max. = 1509); out of these, 8.86% were primary school students (participants in seven studies) and the rest, 91.14%, were secondary school students. In total, five of the studies analyzed had all-girl samples, e.g., [35,36,37,38,39] and one of them was aimed at studying academic procrastination in girls with learning disabilities, e.g., [38] (see Figure 4 for details).

**Figure 5:**
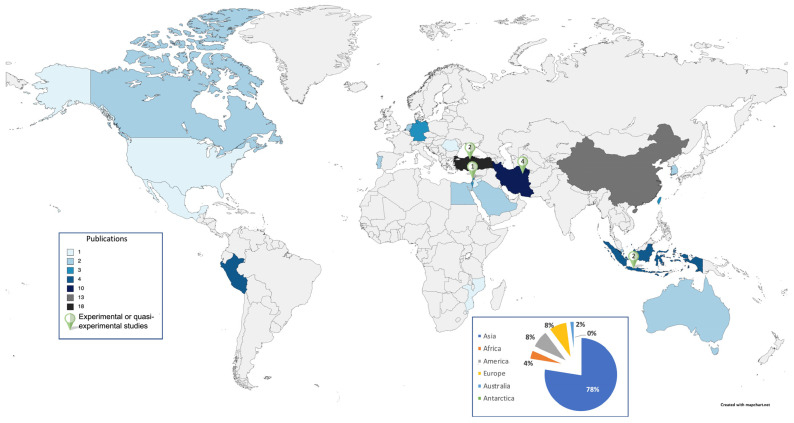
Geographical distribution of samples in studies on academic procrastination in children and adolescents. Note: It should be highlighted that the number of publications does not correspond to the final count due to the presence of a cross-cultural study which includes two different countries.

Regarding the geographical distribution of the samples, Figure 5 specifies the number of publications in each of the continents and countries. Moreover, it lists the countries in which experimental and quasi-experimental studies have been carried out.

#### 3.2.2. Instruments for Assessing Academic Procrastination

In Table 2, the 26 different assessment tools used in the retrieved documents are shown. In general, these are specific questionnaires and scales directly applied to children and adolescents (self-reports), although the participation of parents and teachers (hetero reports) is also reported.

The authors indicate that the instruments used were adapted to the language of the sample; however, they are not questionnaires created specifically for children and adolescents, but mostly validated scales in university students that are also applied in individuals under 18 years old.

The most used instrument is the Procrastination Assessment Scale-Student/PASS, but typically only the first part of the scale, which is designed to measure the frequency of procrastination, is utilized.

#### 3.2.3. Methodology of the Studies and Type of Design

In 97.47% of the studies, a quantitative methodology was employed, while 2.53% utilized qualitative or mixed method approaches. Regarding the design of quantitative studies, 86.07% were ex post facto designs (with an average sample size of 458.64 students), encompassing correlational or group comparison analyses. Additionally, 11.39% of the studies were experimental and quasi-experimental designs, including six randomized controlled trials (RCTs), two randomized non-controlled trials (RNCTs), and one pre-experimental study, with an average sample size of 197.5 students (see Figure 4). Within the ex post facto studies, correlation and regression analyses were predominantly observed, with a recent emergence of analyses based on structural equation modeling (SEM) in recent years.

Regarding studies that incorporate qualitative methodology, they have focused on children’s opinions about behaviors related to procrastination. In one of them [59], children were asked about the reasons for procrastination, strategies to avoid it, and suggestions to reduce this behavior. In this sense, boys mentioned playing computer games as a substitute behavior for studying, while girls preferred activities, such as reading books. The problematic use of mobile phones and the internet has also been studied (e.g., [59]). In this case, students considered access to information as a positive aspect of using the internet, and time loss as a negative aspect.

### 3.3. Content Analysis

#### 3.3.1. Investigated Correlates

The thematic content analysis reveals four main dimensions: Parental and teacher variables (teacher and family), personal variables (sociodemographic, personality, motivation, emotional/affective), mental and well-being (psychopathology, alternative or competitive behaviors, and well-being and quality of life), and variables of the learning cycle (self-regulated learning strategies, performance, and school).

**Figure 6:**
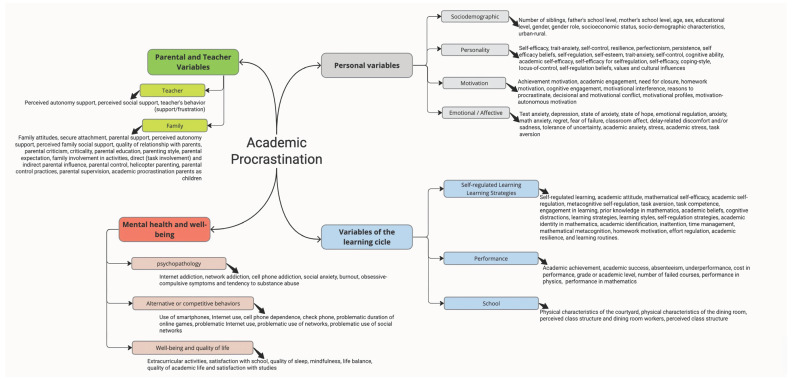
Categories and analyzed variables.

In Figure 6, the set of variables analyzed in the documents (i.e., those that have been measured) is detailed. The most studied variables correspond to dimensions related to the learning cycle, and self-regulated learning occupied the focus of most of this dimension. Thirty-four studies focused on variables related to this process, specifically self-regulated learning, e.g., [60,61,62], time management, e.g., [57,63,64], and inattention or management of distractions, e.g., [62,65]. Regarding previous learning experience and performance [33], we analyzed the role of experience in receiving low grades on exams and assignments and its relationship with academic procrastination. All studies that included performance and academic achievement variables, a total of 22, e.g., [52,66,67] were included in this category.

On the other hand, studies that relate personality variables to academic procrastination are also among the most numerous, with a total of 31, highlighting self-efficacy or self-efficacy beliefs, e.g., [62,68,69,70,71,72], perfectionism, e.g., [57,68,73,74,75], and self-esteem, e.g., [33,67]. Other less common constructs are resilience, locus of control, and persistence.

The relationship between emotional and affective variables with academic procrastination was studied in 11 publications, with test anxiety being the most frequent variable, e.g., [75]; but not only related to evaluation, but also general academic anxiety, anxiety in the study process or for specific subjects, e.g., [32,76].

A specific area of study in this population of children and adolescents is parental support and the development of autonomy; a total of 18 publications analyzed variables related to the quality of parental relationships, parenting style, and parental involvement in education, e.g., [72,76,77,78,79], attention to the promotion of autonomy or excessive parental control, e.g., [78,79].

Alternative behaviors to studying, such as mobile phone use or internet browsing, have also been of interest to researchers, with nine publications dedicated to exploring the relationship between academic procrastination and these types of behaviors, e.g., [14,16,80,81].

Among the retrieved documents, there are also publications that study academic procrastination in the context of psychopathology, such as three studies that analyze procrastination behavior associated with addictive behaviors related to the internet, use of social networks, and electronic devices, e.g., [82,83,84], social anxiety disorder [80,82], and obsessive-compulsive symptoms [70].

#### 3.3.2. Types of Interventions

Regarding the retrieved experimental studies and considering the intervention area, a total of nine studies were selected. These studies can be classified, according to their practical perspective, as follows: Healthcare (1); educational (2); psychoeducational (4); psychotherapeutic (1); and mixed (1).

From a broad socio-sanitary perspective, with an emphasis on child health within social, family, and educational context, Kocoglu and Emiroğlu [85] studied the impact of a school nursing service on the academic performance of 4th-grade students. The authors reported an increase in academic performance, while decreasing absenteeism and academic procrastination behaviors.

On the other hand, from an educational framework, Santyasa et al. [56] compared two teaching methods in two groups of students: A project-based learning (PjBL) model and a direct instruction (DI) model. They found that the project-based method improved academic performance, but this occurred primarily in the group of students with low levels of academic procrastination. From the same perspective, Dong and Izadpanah [35] compared the effect of corrective feedback after formative feedback in an experimental group with control group (women), obtaining improvement in academic resilience, educational belonging, and reduction in procrastination compared to the control group.

From a psycho-educational framework, Ghadampour [86] trained a group of female students in learning strategies (cognitive and metacognitive): The experimental group showed a reduction in procrastination and an increase in self-efficacy that was maintained during the follow-up phase, with significantly differential effects compared to the control group without intervention. Additionally, Motie [87] reported a study based on training in self-regulated learning strategies (boys) in an experimental group design with a control group, which resulted in a decrease in procrastination behavior in the experimental group. Moreover, from the psycho-educational framework, Jaradat [88] reported a study based on three groups: One assigned to cognitive therapy, study skills counseling, and a control group (waiting list). The two experimental groups improved satisfaction with studying in the post-test. The study skills counseling group showed a greater reduction in academic procrastination than the CT group, which decreased exam anxiety but not procrastination. Finally, Yildiz and Iskender [89] implemented a psycho-educational program aimed at secure attachment style in a control group (13 years old) that decreased intolerance of uncertainty and academic procrastination in the experimental group, compared to the control group without intervention, with effects that persisted after the program ended.

From a clinical psychological perspective, Lubis and Djuwita [37] intervened online and in a group format, through a CBT program aimed at academic procrastination, with five girls during the COVID-19 pandemic, with a pre-post and follow-up design. The authors reported that the intervention decreased levels of procrastination.

From a mixed perspective, Kheirkhah [36] trained girls in self-efficacy and learning strategies during eight sessions. The intervention significantly decreased procrastination compared to the control group without intervention.

## 4. Discussion

The aim of this work has been to carry out a scoping review of the empirical publications focused on academic procrastination in children and adolescents.

Regarding the first objective, which aims to identify the production and evolution of publications on academic procrastination in primary and secondary education, the results indicate a lack of significant interest from researchers in this topic.

In contrast to academic procrastination in university students [17,29,90,91,92], there has been a clear increase in the number of publications since 2020. In fact, 45.5% of the retrieved records are dated after this year. It is worth noting that 2020 was the year of the pandemic, with widespread confinement and the consequent implementation of online education systems at all levels [93], as well as a significant rise in the use of social media. Therefore, it is highly likely that this newfound interest has been influenced by the circumstances that occurred. Only time will tell if this trend continues in the coming years.

Regarding the second objective of identifying the common methodological characteristics of the studies, the most relevant data showed that academic procrastination has been predominantly studied in secondary education (92%). In terms of sample sizes, it is noteworthy that 90% of the studies were conducted with very large (>1000) or large (>350 students) samples. It is striking that only 11.4% of the studies were experimental in nature; the majority of the research has focused on exploring relationships between variables. Hopefully, in the future, there will be initiatives aimed at detecting and specifically intervening in this age group.

A high proportion of the retrieved studies came from Eastern countries, with a very limited presence of studies from Europe and America. These findings are similar to those reported by Lu et al. [40], in their meta-analysis on sociodemographic characteristics and procrastination, the authors found a large sample of studies with Chinese participants. On the other hand, Mann [94], in an exploratory study on cultural differences in decision-making in six cultures (USA, Australia, New Zealand, Japan, Hong Kong, and Taiwan), found that Asian students scored higher in academic procrastination compared to Western students, which could justify the differential interest shown by Eastern researchers compared to Western ones. This imbalance is even more pronounced if we consider that there is an Anglo-Saxon bias in the location of international literature in this review.

Within the methodological aspects, the analysis of the measurement instruments used to assess academic procrastination in students under 18 years revealed that many of the scales commonly used have not been specifically created for this age group [40]. This can represent a methodological limitation and a challenge when interpreting results. This highlights the need to reflect on more appropriate evaluation methods for children. For example, the development of observational scales could be a promising approach.

Recording the third objective, which aimed to conduct a content analysis to categorize the correlates investigated in relation to academic procrastination and determine the types of interventions reported, four topics were identified. The content analysis yielded four topics, including constructs related to self-regulated learning [95,96] and a range of personality, motivational, e.g., [97], and emotional variables [98,99], which were expected to be present. Additionally, a third topic emerged concerning the relationships established with significant individuals and the influence of specific variables, such as parenting style, excessive demands, and overprotection on the development of autonomy and self-control in children. Furthermore, an existing but less developed topic pertains to the relationship between procrastination and physical and mental health variables. While this is an emerging area, likely linked to post-pandemic studies, it requires greater attention. We cannot forget that this relationship has been well-studied in academic procrastination in adults [22,82].

The intervention studies reviewed, despite being scarce, show great methodological diversity in terms of sample size and characteristics (e.g., exclusively girls), making it impossible to draw conclusions on effectiveness. This, however, goes beyond the scope of this review. Nevertheless, given the insufficient number of studies found and the methodological problems detected, we can conclude that we are not yet close to conducting a meta-analysis focused exclusively on this age group and being able to answer specific questions.

Building on the previous discussion, we cannot only consider methodological issues related to assessing procrastination in children or the variables that explain the complexity of the construct, but also broader questions such as: What are the limits of the concept of procrastination in childhood? What is the alternative or competing behavior to completing necessary and obligatory tasks in children? Can we say that a child who is playing rather than doing their homework is procrastinating? On the other hand, should we ask ourselves if an inactive child who is not playing is also procrastinating? We believe that one of the important tasks of childhood is the development of play, pleasure, and a sense of fun, and we wonder when the “obligation of tasks” begins to give way to procrastination. The role of school in this regard seems to be very relevant, and it is logical to think that they have the possibility to detect and intervene early.

Is there procrastination in a childhood free of mandatory tasks? We can evoke hundreds of images of children demanding constant activity and insatiable curiosity. At what point, and due to what factors, does learning—or its tasks—become “procrastinatable”?

The analyzed studies do not address these issues. They also do not study, longitudinally, the transition through the stages or levels of study and the adaptation to the different changes and growing demands for autonomy, and how they impact procrastination behavior. In studies with university students, the relationship between academic procrastination and the change in the educational system has been discussed: The autonomy granted by higher education can be a stressor or a promoter of excessive flexibility, which in turn, may favor the emergence of procrastination behavior. In this regard, special attention has been paid to integration during the first year, due to the high dropout rates observed in this course and its relationship with academic self-efficacy, e.g., [97], use of self-regulation strategies, academic engagement, e.g., [100], and academic burnout, e.g., [101]. It is valid to question whether throughout primary and secondary education, the processes of change and transition between these systems present, in a similar way to what has been studied in university samples, critical moments related to the loss of self-regulatory processes and the consequent emergence of greater procrastination. Therefore, longitudinal and long-term studies are necessary.

This scoping review has inherent limitations associated with its scope and breadth, as it aims to provide a comprehensive overview of the literature rather than conduct a detailed assessment of individual study biases. Therefore, the assessment of individual study bias has not been conducted in this review. It is important to acknowledge these limitations when interpreting the findings and to consider them in future research.

## 5. Conclusions

In this study, we have found that there is a large proportion of publications reporting on the relationship between academic procrastination and personality, motivational, and self-regulation variables (both of learning and emotion) in childhood and adolescence. However, our analysis of the classification categories has allowed us to identify gaps in the literature, such as the need to define and operationalize the construct of procrastination in this age group and to develop appropriate assessment tools and techniques. Overall, it is not clear how prevalent the problem of academic procrastination is in this age group, while it is well-known to be a major problem in higher education, with broad consequences for psychological emotional and physical health, academic performance, and well-being.

Based on the studies analyzed here, the authors argue for the need to clarify, through different methodological strategies, the role of promoting autonomy, developing self-regulation and self-control in childhood, and how they relate to parenting styles, educational models, and teaching strategies. Emphasizing the possibility of early detection and intervention methods may pave the way for reducing the high prevalence of procrastination in university students.

The main conclusions are as follows:

- Addressing academic procrastination in children and adolescents should consider both individual and contextual factors, as well as appropriate interventions;
- There is a need for the development of more appropriate assessment tools to measure academic procrastination in children and adolescents, considering their specific developmental characteristics;
- The prevalence of academic procrastination in this population is still understudied, highlighting a research gap that requires further attention;
- In summary, further research and interventions are necessary to improve the understanding and management of academic procrastination in children and adolescents.

In order to enhance knowledge in this field, it is suggested to develop methodological improvements (such as the use of appropriate instruments, operationalization of the target behaviors under study, and high-quality research reporting) and promote the implementation of policies that provide specific financial support to foster collaboration between institutions and different knowledge areas.

## Figures and Tables

**Table 1:** Inclusion and exclusion criteria.

Criteria	Inclusion	Exclusion
Documents (d)	Journal articles, books, book chapters, and doctoral theses	Magazine articles, editorials, conferences, etc.
Concept (Co)	Academic procrastination	The rest
Context (Co)	Academic context	Other contexts (e.g., general, work or health procrastination)
Studies (s)	Empirical studies	Theoretical reviews and case studies
Participants (p)	Students under 18 years	University students or community samples
Evaluation (e)	Behavioral or reported procrastination measures (i.e., self-reports or hetero reports)	Single-item instruments or unstructured instruments

**Table 2:** Recount of instruments used.

Instrument	Count
Procrastination Assessment Scale-Student—PASS (Solomon and Rothblum, 1984 [40])	23
Academic Procrastination Scale—AP-S (Çakıcı, 2003 [41])	10
Tuckman Procrastination Scale—TPS (Tuckman, 1991 [40])	8
Aitken Procrastination Inventory—API (Aiken, 1982 [42])	5
General Procrastination Scale—GPS (Lay, 1986 [43])	5
Escala de Procrastinación Académica—EPA (Busko, 1998 [44])	4
Academic Procrastination Questionnaire (Huang, 2009 [45])	2
Scale developed by authors (Dietz et al., 2007 [46])	3
Academic Procrastination Scale—APS-S (McCloskey, 2011 [47])	2
Academic Procrastination Inventory for Middle School Students—API-MSS (Zuo, 2020 [48])	1
Academic Procrastination Questionnaire (Ran, H., 2010 [49])	1
Academic Procrastination Scale—MSLQ (Lay and Silverman, 1996 [50])	1
Academic Procrastination Scale—APS (Lay, 1986 [43])	1
Academic Procrastination Student Form—APS (Milgram and Amir, 1998 [32])	1
Academic Procrastination Survey (Savari, K., 2011 [51])	1
Cuestionario de Procrastinación en el Estudio (CPE; Rosário et al., [52])	3
Irrational Procrastination Scale—IPS (Steel, 2010 [53])	1
Melbourne Decision Making Questionnaire (five items procrastination) (Mann et al., 1997 [54])	1
Scale developed by authors (Depreeuw and Lens, 1998 [55])	2
Scale developed by authors (Santyasa et al., 2020 [56])	1
Scale developed by authors (Shih, 2016 [57])	
Scale developed by authors (Ocak and Karatas, 2019 [58])	1
Academic Procrastination Scale—DPS (Ferrari et al., 1995 [13])	1
Total	79

## Data Availability

Data can be accessed via contacting the authors upon approval.

## Appendix A

**Table A1:** Preferred Reporting Items for Systematic reviews and Meta-Analyses extension for Scoping Reviews (PRISMA-ScR) Checklist [25].

Section	Item	PRISMA-ScR Checklist Item	Reported on Page # (Number)
Title			
Title	1	Identify the report as a scoping review.	1
Abstract			
Structured summary	2	Provide a structured summary including, as applicable: Background, objectives, eligibility criteria, sources of evidence, charting methods, results, and conclusions that relate to the review question(s) and objective(s).	1
Introduction			
Rationale	3	Describe the rationale for the review in the context of what is already known. Explain why the review question(s)/objective(s) lend themselves to a scoping review approach.	1–2
Objectives	4	Provide an explicit statement of the question(s) and objective(s) being addressed with reference to their key elements (e.g., population or participants, concepts, and context), or other relevant key elements used to conceptualize the review question(s) and/or objective(s)).	2
Methods			
Protocol, and registration	5	Indicate if a review protocol exists, if and where it can be accessed (e.g., web address), and, if available, provide registration information including registration number.	3
Eligibility criteria	6	Specify the characteristics of the sources of evidence (e.g., years considered, language, publication status) used as criteria for eligibility, and provide a rationale.	3, 6
Information sources	7	Describe all information sources (e.g., databases with dates of coverage, contact with authors to identify additional sources) in the search, as well as the date the most recent search was executed.	4
Search	8	Present the full electronic search strategy for at least one database, including any limits used, in order that it could be repeated.	4
Selection of sources of evidence	9	State the process for selecting sources of evidence (i.e., screening, eligibility) included.	5
Data charting process	10	Describe the methods of charting data from the included sources of evidence (e.g., piloted forms, forms that have been tested by the team before their use, whether data charting was carried out independently, in duplicate) and any processes for obtaining and confirming data from investigators.	5
Data items	11	List and define all variables for which data were sought and any assumptions and simplifications made.	5
Critical appraisal of individual sources of evidence	12	If performed, provide a rationale for conducting a critical appraisal of included sources of evidence, describe the methods used, and how this information was used in any data synthesis (if appropriate).	N/A
Summary measures	13	Not applicable for scoping reviews.	
Synthesis of results	14	Describe the methods of handling and summarizing the data that were charted.	5
Risk of bias across studies	15	Not applicable for scoping reviews.	
Additional analyses	16	Not applicable for scoping reviews.	
Results			
Selection of sources of evidence	17	Give numbers of sources of evidence screened, assessed for eligibility, and included in the review, with reasons for exclusions at each stage, ideally using a flow diagram.	6
Characteristics of sources of evidence	18	For each source of evidence, present characteristics for which data were charted and provide the citations.	6, 7, 8, 10
Critical appraisal within sources of evidence	19	If performed, present data on critical appraisal of included sources of evidence (see item 12).	
Results of individual sources of evidence	20	For each included source of evidence, present the relevant data that were charted that relate to the review question(s) and objective(s).	Appendix B
Synthesis of results	21	Summarize and/or present the charting results as they relate to the review question(s) and objective(s).	5, 6, 7, 8, 9
Risk of bias across studies	22	Not applicable for scoping reviews.	
Additional analyses	23	Not applicable for scoping reviews.	
Discussion			
Summary of evidence	24	Summarize the main results (including an overview of concepts, themes, and types of evidence available), explain how they relate to the review question(s) and objectives, and consider the relevance to key groups.	11–12
Limitations	25	Discuss the limitations of the scoping review process.	13
Conclusions	26	Provide a general interpretation of the results with respect to the review question(s) and objective(s), as well as potential implications and/or next steps.	13
Funding			
Funding	27	Describe sources of funding for the included sources of evidence, as well as sources of funding for the scoping review. Describe the role of the funders of the scoping review.	14

## Appendix B

**Table A2:** Summary of Scoping Review.

Bibliometric Information			Sample			Design
Author	Year	Doc. Type	Edu Level	N	Country	
Milgram et al. [102]	1995	Article	SS	195	ISR	EPF
Owens and Newbegin [67]	1997	Article	PS	418	AUS	EPF
Milgram and Toubiana [32]	1999	Article	SS	245	ISR	EPF
Owens and Newbegin [33]	2000	Article	SS	380	AUS	EPF
Davis [74]	2001	Thesis	SS	284	USA	EPF
Nadeau et al. [103]	2003	Article	SS	100	CAN	EPF
Jaradat [88]	2004	Thesis	SS	729	JOR	RCT
Dietz and et al. [46]	2007	Article	SS	704	DEU	EPF
Rosário et al. [75]	2008	Article	SS	533/796	PRT	EPF
Klassen and Kuzucu [34]	2009	Article	SS	508	TUR	EPF
Klassen et al. [104]	2009	Article	SS	612	CAN, SGP	EPF
Uzun Özer [105]	2009	Article	SS	223	TUR	EPF
Rosário et al. [75]	2009	Article	SS	580/809	PRT	EPF
Al-Attiyah [106]	2010	Article	PS	538	QAT	EPF
Kalafat et al. [70]	2010	Article	SS	285	TUR	EPF
Kuhnle et al. [107]	2011	Article	SS	348	DEU	EPF
Uzun Özer and Ferrari [108]	2011	Article	SS	214	TUR	EPF
Liu and Lu [109]	2011	Article	SS	712	CHN	EPF
Hofer et al. [110]	2012	Article	SS	697	DEU	EPF
Motie et al. [111]	2012	Article	SS	250	IRN	EPF
Mih [77]	2013	Article	SS	189	ROU	EPF
Motie et al. [87]	2013	Article	SS	66	IRN	RCT
Bong et al. [68]	2014	Article	SS	306	KOR	EPF
Katz et al. [71]	2014	Article	PS	171	ISR	EPF
Tang et al. [72]	2014	Article	SS	460	CHN	EPF
Hong et al. [78]	2015	Article	SS	597	TWN	EPF
Jahromi et al. [112]	2015	Article	SS	268	IRN	EPF
Gazidari et al. [113]	2016	Article	SS	311	IRN	EPF
Ng [114]	2016	Article	SS	442	SGP	EPF
Shih [57]	2016	Article	SS	405	TWN	EPF
Zohreyi et al. [39]	2016	Article	SS	210	IRN	EPF
Chen and Han [115]	2017	Article	SS	577	CHN	EPF
Günlü and Ceyhan [59]	2017	Article	SS	1088	TUR	EPF
Kocoglu and Emiroglu [85]	2017	Article	PS	31	TUR	RNCT
Shih [63]	2017	Article	SS	405	TWN	EPF
Yang et al. [116]	2017	Article	SS	360	CHN	EPF
Borekci and Uyangor [76]	2018	Article	SS	496	TUR	EPF
Jiang et al. [69]	2018	Article	SS	848	KOR	EPF
Korkmaz et al. [117]	2018	Article	SS	167	TUR	EPF
Mostafa [38]	2018	Article	SS	100	EGY	EPF
Ziegler and Opdenakker [62]	2018	Article	SS	566	NLD	EPF
Ghadampour and Beiranvand [86]	2019	Article	SS	30	IRN	RCT
Macias and Nevarez [118]	2019	Article	SS	300	MEX	EPF
Ahmadi et al. [119]	2020	Article	SS	149	IRN	EPF
Sula Atas and Kumcagiz [120]	2020	Article	SS	602	TUR	EPF
Durak [80]	2020	Article	SS	451	TUR	EPF
Eissa and Khalifa [121]	2020	Article	SS	228	EGY	EPF
Gündüz [122]	2020	Article	SS	358	TUR	EPF
Lenggono and Tentama [123]	2020	Article	SS	60	IDN	EPF
Saad [60]	2020	Article	SS	68	EGY	EPF
Santyasa et al. [56]	2020	Article	SS	124	IDN	RNCT
Tras and Gökçen [1]	2020	Article	SS	599	TUR	EPF
Trujillo-Chumán and Noé-Grijalva [124]	2020	Article	SS	366	PER	EPF
Zhen et al. [65]	2020	Article	SS	1505	CHN	EPF
Çam and Ögülmüs [73]	2021	Article	SS	1068	TUR	EPF
Fulano et al. [125]	2021	Article	SS	1000	MOZ	EPF
Hong et al. [81]	2021	Article	SS	633	CHN	EPF
Khalifa [83]	2021	Article	SS	250	SAU	EPF
Kheirkhah [36]	2021	Article	SS	559	IRN	RCT
Niu et al. [126]	2021	Article	SS	623	CHN	EPF
Opdenakker [127]	2021	Article	SS	687	NLD	EPF
Quispe et al. [61]	2021	Article	SS	36	PER	EPF
Rezaei and Zebardast [128]	2021	Article	SS	350	IRN	EPF
Selçuk et al. [129]	2021	Article	SS	1000	TUR	EPF
Wang [130]	2021	Article	SS	566	CHN	EPF
Xue et al. [66]	2021	Article	PS	338	CHN	EPF
Yang et al. [131]	2021	Article	SS	353	CHN	EPF
Yildiz and Iskender [89]	2021	Article	SS	1068	TUR	RCT
Yu et al. [132]	2021	Article	SS	24	CHN	EPF
Asmali and Sayin [133]	2022	Article	PS	245	TUR	EPF
Chaji and Ebrahimpour [134]	2022	Article	PS	662	CHN	EPF
Dong and Izadpanah [35]	2022	Article	SS	5	IRN	RCT
Galindo-Contreras and Olivas-Ugarte [135]	2022	Article	SS	256	PER	EPF
Gezgin [84]	2022	Article	SS	300	TUR	EPF
Lubis and Djuwita [37]	2022	Article	SS	1255	IDN	PE
Muarifah et al. [15]	2022	Article	SS	429	IDN	EPF
Salluca et al. [14]	2022	Article	SS	370	PER	EPF
Üztemur and Dinç [16]	2022	Article	SS	136	TUR	EPF
Zhao et al. [79]	2022	Article	SS	514	CHN	EPF

Note: Edu Level (Educational Level): PS (Primary School) and SS (Secondary School). Country: AUS (Australia), CAN (Canada), CHN (China), DEU (Germany), EGY (Egypt), IDN (Indonesia), IRN (Iran), ISR (Israel), JOR (Jordan), KOR (South Korea), MEX (Mexico), MOZ (Mozambique), NLD (Netherlands), PER (Peru), PRT (Portugal), QAT (Qatar), ROU (Romania), SAU (Saudi Arabia), SGP (Singapore), TUR (Turkey), TWN (Taiwan), and USA (United States). Design: EPF (Ex Post Facto), PE (Pre-Experimental study), RCT (Randomized Controlled Trial), and RNCT (Randomized non-Controlled Trial).
